# Melatonin promotes bone marrow mesenchymal stem cell osteogenic differentiation and prevents osteoporosis development through modulating circ_0003865 that sponges miR-3653-3p

**DOI:** 10.1186/s13287-021-02224-w

**Published:** 2021-02-25

**Authors:** Xudong Wang, Taiqiu Chen, Zhihuai Deng, Wenjie Gao, Tongzhou Liang, Xianjian Qiu, Bo Gao, Zizhao Wu, Jincheng Qiu, Yuanxin Zhu, Yanbo Chen, Zhancheng Liang, Hang Zhou, Caixia Xu, Anjing Liang, Peiqiang Su, Yan Peng, Dongsheng Huang

**Affiliations:** 1 Department of Orthopedics, Sun Yat-sen Memorial Hospital of Sun Yat-sen University, #107 West Yan Jiang Road, Guangzhou, Guangdong 510120 China; 2 Department of Orthopedics, the First Affiliated Hospital of Sun Yat-sen University, Guangzhou, Guangdong China; 3 Guangdong Provincial Key Laboratory of Orthopedics and Traumatology, the First Affiliated Hospital of Sun Yat-sen University, Guangzhou, Guangdong China; 4 Department of Orthopedics, the Third Affiliated Hospital of Sun Yat-sen University, Guangzhou, Guangdong China; 5 Research Centre for Translational Medicine, the First Affiliated Hospital of Sun Yat-sen University, Guangzhou, Guangdong China

**Keywords:** Melatonin, Osteogenic differentiation, BMSCs, Osteoporosis, circ_0003865, miR-3653-3p

## Abstract

**Background:**

Little is known about the implications of circRNAs in the effects of melatonin (MEL) on bone marrow mesenchymal stem cell (BMSC) osteogenic differentiation and osteoporosis (OP) progression. The aim of our study was to investigate circRNAs in MEL-regulated BMSC differentiation and OP progression.

**Methods:**

BMSC osteogenic differentiation was measured by qRT-PCR, western blot (WB), Alizarin Red, and alkaline phosphatase (ALP) staining. Differential circRNA and mRNA profiles of BMSCs treated by MEL were characterized by deep sequencing, followed by validation using RT-PCR, Sanger sequencing, and qRT-PCR. Silencing and overexpression of circ_0003865 were conducted for functional investigations. The sponged microRNAs and targeted mRNAs were predicted by bioinformatics and validated by qRT-PCR, RNA pull-down, and dual-luciferase reporter assay. The function of miR-3653-3p and circ_0003865/miR-3653-3p/growth arrest-specific gene 1 (GAS1) cascade was validated for the osteogenic differentiation of BMSCs by CCK-8, qRT-PCR, WB, Alizarin Red, and ALP staining. The effects of circ_0003865 on OP development were tested in murine OP model.

**Results:**

MEL promoted osteogenic differentiation of BMSCs. RNA sequencing revealed significant alterations in circRNA and mRNA profiles associated with multiple biological processes and signaling pathways. Circ_0003865 expression in BMSCs was significantly decreased by MEL treatment. Silencing of circ_0003865 had no effect on proliferation while promoted osteogenic differentiation of BMSCs. Overexpression of circ_0003865 abrogated the promotion of BMSC osteogenic differentiation induced by MEL, but proliferation of BMSCs induced by MEL had no change whether circ_0003865 was overexpression or not. Furthermore, circ_0003865 sponged miR-3653-3p to promote GAS1 expression in BMSCs. BMSC osteogenic differentiation was enhanced by miR-3653-3p overexpression while BMSC proliferation was not affected. By contrast, miR-3653-3p silencing mitigated the promoted BMSC osteogenic differentiation caused by circ_0003865 silencing, but had no effect on proliferation. Finally, circ_0003865 silencing repressed OP development in mouse model.

**Conclusion:**

MEL promotes BMSC osteogenic differentiation and inhibits OP pathogenesis by suppressing the expression of circ_0003865, which regulates GAS1 gene expression via sponging miR-3653-3p.

## Introduction

Osteoporosis (OP) is a common skeletal metabolic disorder hallmarked by a decrease in bone mineral density (BMD) and deterioration of bone microarchitecture. This frequently leads to a significant elevation susceptibility to fracture because of increased bone fragility [1, 2]. Osteoporotic fractures in the forearm, hip, and lumbar spine were reported to be associated with high morbidity and mortality, especially in the aged population and postmenopausal women [1]. Multiple heritable and nonheritable risk factors are involved in primary OP development, including aging, sex steroid deficiency, oxidative stress genetic mutations, and lifestyle-related factors such as physical inactivity, diet, and alcohol abuse [1]. Concerning pathogenesis, the development of OP may result from inadequate bone formation during growth, excessive bone resorption, and bone remodeling deregulation [2]. The maintenance of bone homeostasis largely depends on the coordinated genesis and apoptosis of osteoclasts and osteoblasts, and the decline and dysfunction of osteoblasts have been established as major pathogenic mechanisms underlying OP development [3, 4]. Although remarkable advances have been achieved during the past decades in OP treatment through the application of estrogen, bisphosphonates, and calcitonin, their overall efficacies remain limited because of multiple side-effects, high expense, and long treatment courses [5]. Recently, the transplantation of bone marrow mesenchymal stem cells (BMSCs) represents a promising strategy for treating OP because of their pluripotent potential [6]. Such treatments are urgently needed to effectively promote BMSC osteogenic differentiation toward osteoblasts.

Melatonin (MEL), alternatively known as 5 methoxy-N-acetyltryptamine, is a key indole hormone primarily secreted by pinealocytes in the pineal glands of mammals and was first characterized in bovine pineal tissues in 1958 [7, 8]. As an amphiphilic chemical messenger, MEL has been widely implicated in various physiological processes, such as circadian and seasonal timing, glycemic control and energy metabolism, neural protection and neuroplasticity, sleep and wake cycle control, immune system function, and oxygen and nitrogen reactive species scavenging as a natural antioxidant [7]. Furthermore, MEL has also been reported to be an essential regulator of bone metabolism processes including bone formation and resorption, bone matrix mineralization, and osteoblast and osteoclast activities [9]. For example, the stimulating effects of MEL on osteoblast proliferation and differentiation have been verified by extensive research under different conditions [9–11]. Contrarily, MEL may suppress the function of osteoclasts by upregulating a section of calcitonin by osteocytes [12]. Importantly, recent studies have shown that MEL promotes bone formation and alleviates bone destruction in a mouse OP model induced by retinoic acid. This effect is mediated by its reduction of oxidant levels and modulation of extracellular signal-regulated kinase and nuclear factor kappa B signaling pathways [13]. Furthermore, MEL maintains BMSC stemness and promotes the osteoblast differentiation and osteogenesis of BMSCs [14–16]. However, the mechanisms underlying MEL-regulated BMSC differentiation and OP development remain poorly understood.

Circular RNAs (circRNAs) are a large set of newly characterized non-coding RNA molecules that are covalently closed and in single-stranded form. They are synthesized through the back-splicing of pre-mRNAs (precursor mRNA) encoded by eukaryotic genes [17]. Recent reports have demonstrated that circRNAs are broadly expressed in distinct tissues and cell types to modulate functional gene expression, mainly by sponging microRNA (miRNA), regulating transcription, and even influencing the pre-mRNA splicing process [17]. Because of their significant impact on gene expression, the dysregulated expression of circRNA profiles has been reported to be closely associated with the pathogenesis of various human disorders, such as cancer, neurological diseases, atherosclerotic vascular disease, and cardiovascular disorders [18]. The expression of circ_0002060 and other circRNAs have been suggested to be potential markers for OP diagnosis because of their correlation with OP pathogenesis [19, 20]. Furthermore, circRNA_0016624 was recently shown to regulate the progression of postmenopausal OP by sponging miR-98 to modulate the expression of the *BMP2* (bone morphogenetic protein 2) gene [19]. Similarly, OP induced by glucocorticoids may be alleviated by circRNA_0006393 by sponging miR-145-5p to elevate forkhead box O1 (*FOXO1*) gene expression [21]. Additionally, the proliferation and osteoblast differentiation of BMSCs and their effects on osteogenesis were also recently reported to be modulated by circRNAs [22]. However, little is known about the implications of circRNAs in the effects of MEL on BMSC osteogenic differentiation and OP progression.

In the present study, we analyzed MEL-induced alterations of circRNA profiles in BMSCs undergoing osteogenic differentiation through large-scale deep RNA sequencing, followed by elucidation of the roles of circ_0003865 in regulating BMSC osteogenic differentiation and OP development using both cell and animal models. These results provide novel insights into the BMSC differentiation-regulating functions of MEL associated with OP pathogenesis, which may also provide novel biomarkers for OP diagnosis and treatment.

## Material and methods

### Human BMSC isolation and culture

The human BMSCs used in the present study were isolated from bone marrow tissues collected from healthy volunteers as previously described [23–25]. Briefly, 8 ml of bone marrow tissue from each volunteer was first washed with 12 ml of pre-chilled phosphate-buffered saline (PBS) and separated by density gradient centrifugation (500 g; 20 min) using Lymphoprep medium (Axis-Shield, Oslo, Norway). The interface liquid layer containing the mononuclear cells was carefully collected, washed with PBS, and resuspended in low-glucose Dulbecco’s modified Eagle’s medium (DMEM; Thermo Fisher Scientific) containing 10% fetal bovine serum (FBS; Gibco). After culturing at 37 °C in a humidified chamber supplied with 5% CO_2_ for 48 h, non-adherent cells were removed by changing the culture medium. The remaining cells were cultured at 37 °C under normal cell culture conditions with the renewal of DMEM medium every 3 days and passaged when cell confluence reached over 80%. After three successive passages, the isolated BMSCs were used for various assays. The research was approved in advance by the Ethical Committee of Sun Yat-sen University. Written informed consent was obtained from each volunteer.

### Osteogenic differentiation induction and treatment

Human BMSCs were cultured in six-well plates at 37 °C until cell confluency reached approximately 85%. The cells were then subjected to induction of osteogenic differentiation by culturing in adult BMSC osteogenic differentiation medium (#HUXMA-90021; Cyagen Biosciences, Guangzhou, China) according to the manufacturer’s instructions. The culture medium was renewed every 3 days. The human BMSCs were then incubated with 100 μmol/L MEL (#M5250-1G; Sigma Aldrich), which was added to the osteogenic differentiation medium. The 293T human embryonic kidney cell line (#SCSP-502) was purchased from the Cell Bank of the Chinese Academy of Sciences (Shanghai, China) and cultured at 37 °C in DMEM containing 10% FBS and supplied with 5% CO_2_.

### Cell transfections and infection

The sequences for the siRNA targeting circ_0003865 were sense: 5′-AGUUACACAGAUUCAGAUCCAUU-3′, antisense: 5′-AAUGGAUCUGAAUCUGUGUAACU-3′; its negative control, sense: 5′-UUCUCCGAACGUGUCACGUTT-3′, antisense: 5′-ACGUGACACGUUCGGAGAATT-3′; the miR-3653-3p mimics, sense: 5′-CUAAGAAGUUGACUGAAG-3′, antisense: 5′-UCAGUCAACUUCUUAGUU-3′; and mimics NC (the same as negative control); the miR-3653 inhibitors: 5′-CUUCAGUCAACUUCUAG-3′ and inhibitor NC: 5′-CAGUACUUUUGUGUAGUACAA-3′. All oligos were synthesized by the GenePharma Biotech company (Shanghai, China). As designated, the above sequences were transfected into human BMSCs using the Lipofectamine TM 2000 transfection kit (Invitrogen, USA) according to the manufacturer’s instructions. For overexpression of circ_0003865, the sequences of circ_0003865 were synthesized and ligated into the LV5 plasmid to construct the recombinant LV5-circ_0003865 vector. LV5 and LV5-circ_0003865 lentiviruses were packaged by the Vigene Biosciences company (Jinan, Shandong, China) and infected into BMSCs according to the manufacturer’s instructions.

### qRT-PCR and RT-PCR

Total RNA samples from cultured BMSCs or mouse tissues were prepared using the Total RNA Extraction Kit (#17200; AmyJet Scientific, Wuhan, China) according to the manufacturer’s instructions. RNA concentrations were determined by the Nanodrop 2000 instrument (Thermo Fisher Scientific). The cDNA for qRT-PCR was subsequently synthesized from 3 μg total RNA using the Omniscript RT Kit (#205111; Qiagen) as instructed by the manufacturer. Subsequently, the real-time quantitative PCR assay was then conducted using the TransStart® Green qPCR SuperMix kit (#AQ101-01; TransGen Biotech, Beijing, China) according to the manufacturer’s instructions. The final expressional levels of circRNAs, microRNAs, or mRNAs were calculated by the standard 2^−△△Ct^ method based on at least three biological replicates. The covalently closed-loop structures of circRNAs were measured by PCR using divergent primers, whereas opposite-directed primers targeting linear genomic DNA were used as controls. The sequences of all primers used for quantitation are listed in Table 1.

**Table 1:** Sequences of primers used for quantitative PCR

Primer name	Primer sequences (5′–3′)
RUNX2 F	AGAAGGCACAGACAGAAGCTTGA
RUNX2 R	AGGAATGCGCCCTAAATCACT
OPN F	GCGAGGAGTTGAATGGTG
OPN R	GCGAGGAGTTGAATGGTG
ALP F	GAGTCGGACGTGTACCGGA
ALP R	TGCCACTCCCACATTTGTCAC
hsa_circ_0002770-CF1	TGTATCAGGCAGGGGAGAGT
hsa_circ_0002770-CR1	ACACAGAGCCAGGCTTTCAT
hsa_circ_0073244-CF1	CTGGACAAGCAAGGCAAAGT
hsa_circ_0073244-CR1	CTTGGCTCCTTGGGTAATCA
hsa_circ_0003126-CF1	CAAAGCAGTGGGCTCACATA
hsa_circ_0003126-CR1	GGCCTCCAATTCATTCAGTC
hsa_circ_0002867-CF1	ACAGTCCGCAATGCCTTAAA
hsa_circ_0002867-CR1	TTCAAGAGAGCCGTCCAACT
hsa_circ_0008210-CF1	GCTGCATCAAGAAACCCAAG
hsa_circ_0008210-CR1	CAGTTGCTCCACATCTCTGC
hsa_circ_0037026-CF1	CCATTCTCATGCCTTGGTCT
hsa_circ_0037026-CR1	TGGCAGCACTCATTGTTCTC
hsa_circ_0005015-CF1	AGAGCACTGGGACGAAGTGT
hsa_circ_0005015-CR1	AAGCAGCTGTGATTCCAAGG
hsa_circ_0006935-CF1	CCCTGAGTTGGTGCTGAAA
hsa_circ_0006935-CR1	ATGATGGGCTTGGTAGGTGA
hsa_circ_0003865-CF1	ACTTCGTCACGGTGGAAATC
hsa_circ_0003865-CR1	CCCCCAATTTTCACTTGTATG
hsa_circ_0003865-LF1	CAACGCCTGCCCAAAAGAAA
hsa_circ_0003865-LR1	AGTGGTGTCCAACCTGCAAA
H-GAPDH-conver-F	GAGTCAACGGATTTGGTCGT
H-GAPDH-conver-R	GACAAGCTTCCCGTTCTCAG
H-GAPDH-diver-F	TCTGACTTCAACAGCGACAC
H-GAPDH-diver-R	TGACGGTGCCATGGAATTTG
hsa-miR-3653-3p-RT	GTCGTATCCAGTGCAGGGTCCGAGGTATTCGCACTGGATACGACCTTCAG
hsa-miR-3653-3p-F	CTAAGAAGTTGACTGAAG
hsa-miR-4775-RT	GTCGTATCCAGTGCAGGGTCCGAGGTATTCGCACTGGATACGACAGTGAC
hsa-miR-4775-F	TTAATTTTTTGTTTCGGTCACT
hsa-miR-511-5p-RT	GTCGTATCCAGTGCAGGGTCCGAGGTATTCGCACTGGATACGACTGACTG
hsa-miR-511-5p-F	GTGTCTTTTGCTCTGCAGTCA
hsa-miR-6509-3p-RT	GTCGTATCCAGTGCAGGGTCCGAGGTATTCGCACTGGATACGACAAATTA
hsa-miR-6509-3p-F	TTCCACTGCCACTACCTAATTT
hsa-miR-942-5p-RT	GTCGTATCCAGTGCAGGGTCCGAGGTATTCGCACTGGATACGACCACATG
hsa-miR-942-5p-F	TCTTCTCTGTTTTGGCCATGTG
Universe-R	GTGCAGGGTCCGAGGT
GAS1-F	GACCTACTGCGGCAAAGTCT
GAS1-R	GCCATGTTCTCCTTGACCGA
SFRP2-F	GACCATTTCTGCTCCGGGAT
SFRP2-R	CAGCTATCCACTCCTGTGGC
hsa-U6-F	CTCGCTTCGGCAGCACA
hsa-U6-R	AACGCTTCACGAATTTGCGT

### Western blotting

Total protein samples were extracted from cultured BMSCs using the Total Protein Extraction kit (#C006225-0050; Sangon Biotech, Shanghai, China) according to the manufacturer’s instructions. The protein concentrations were determined by the bicinchoninic acid method. Approximately 30 μg of protein was then boiled at 100 °C for 5 min, separated by 12% sodium dodecyl sulfate polyacrylamide gel electrophoresis, and blotted onto polyvinylidene fluoride (PVDF) membranes The membranes were subsequently blocked in 5% lipid-free milk solution, incubated with diluted primary antibodies, washed three times with TBST solution, and incubated with diluted secondary antibodies. Finally, the PVDF membranes were developed with the EasyBlot ECL (enhanced chemiluminescence) kit (#D601039-0050; Sangon Biotech, Shanghai, China) according to the manufacturer’s instructions. At least three biological replicates were conducted for protein quantitation, and GAPDH was used as an internal standard. The antibodies used in the present study included anti-ALP (#ab229126; Abcam), anti-osteopontin (OPN) (#ab8448; Abcam), anti-runt-related transcription factor 2 (RUNX2) (#12556; CST), anti-GAPDH (#ab181602; Abcam), and anti-growth arrest-specific gene 1 (GAS1) (#ab236618; Abcam).

### CircRNA and mRNA profiling

The differential expression of circRNAs and mRNA profiles for human BMSCs between BMSC osteogenic differentiation induction group and BMSC osteogenic differentiation induction with 100 μM MEL treatment at the same time group were determined by the deep RNA sequencing method. Briefly, total RNA samples from each group were prepared using TRIzol reagent (Thermo Fisher Scientific) according to the manufacturer’s instructions, and the concentration of the RNA was measured using the Nanodrop 2000 instrument. RNA integrity was evaluated using the Agilent 2100 Bioanalyzer instrument. Then, the rRNA components were removed from the RNA samples using the RiboMinus™ Eukaryote Kit for RNA-Seq (#A1083708; Thermo Fisher Scientific) according to the manufacturer’s instructions. For circRNA sequencing, the digestion of linear RNAs was done using RNase R. For mRNA sequencing, the eukaryotic mRNA samples were enriched using Oligo-dT beads (Thermo Fisher Scientific) according to the manufacturer’s instructions. An RNA library was generated from the collected RNA samples using the NEBNext Ultra II RNA Library Prep Kit (#E7770S; NEB) according to the manufacturer’s instructions. Subsequently, the RNA library was sequenced using the HiSeq 2000 sequencing system (Illumina, USA).

### Bioinformatics

The clean reads obtained from the RNA sequencing were then aligned with the human reference genome database using Bowtie2 software. The back-splice algorithm was subsequently conducted to select for read junctions. The CIRI software was used to predict and annotate the circRNAs from all mapped reads. The differential expression levels of circRNAs in human BMSCs induced by MEL treatment after osteogenic differentiation induction were determined by calculating the RPKM (mapped back-splicing junction reads per kilobase per million mapped reads) value, which was calibrated to the total read numbers. The significantly differential expression of circRNAs or mRNAs in MEL-treated BMSCs compared with BMSCs was determined using a log2Ratio of > 1 combined with a false discovery rate of < 0.05. The differential expression of circRNAs and mRNAs were further analyzed by hierarchical clustering and scatter plots, which were established using the R software package (Version 0.2.3). Subsequently, the functional categorization of differentially expressed mRNAs based on Gene Ontology (GO) terms was done by searching the Database for Annotation, Visualization, and Integrated Discovery. The enrichment of differentially expressed mRNAs in signaling pathways was done by searching the Kyoto Encyclopedia of Genes and Genomes (KEGG) database (http://www.genome.jp/kegg/). The interaction between circRNA, microRNA, and mRNAs was predicted using String V10 software.

### Cell Counting Kit-8 (CCK-8) assay

After treatment, the proliferation of BMSCs was detected with the CCK-8 kit (#C0037, Beyotime Biotech, Shanghai, China) following the manufacturer’s instructions. Briefly, BMSCs were digested by trypsin (Thermo Fisher Scientific) and then were seeded into a 96-well plate at a density of 4 × 10^3^ cells pre well. After 24 h, 48 h, and 72 h, Cell Counting Kit-8 (CCK-8) was added to detect the proliferation of BMSCs via Elx800 (BioTek, Winooski, Vermont, USA) at 450 nm spectrophotometric.

### Alkaline phosphatase and alizarin red staining

The expression of alkaline phosphatase (ALP) in human BMSCs was detected using the BCIP/NBT Alkaline Phosphatase Color Development Kit (#C3206; Beyotime Biotech) according to the manufacturer’s instructions. Briefly, cell slides were washed three times with PBS solution, fixed in 4% paraformaldehyde, and washed again three times with PBS solution. The slides were then incubated with BCIP/NBT staining solution for 1–2 h at room temperature in darkness, washed twice with distilled water for 3 min. Finally, ALP expression in human BMSCs was observed by gross scanning (Bio-Rad, GS-800, Hercules, CA, USA) and light microscopy (OPTEC, TP510, Chongqing, China). At least three biological replicates were conducted for comparison. To evaluate osteogenic differentiation, BMSCs were also stained with Alizarin Red S reagent (#ST1078-25g; Beyotime Biotech) for 1–5 min at room temperature, as outlined in the manufacturer’s instructions. Quantification of ALP and Alizarin Red S were conducted using ImageJ software (National Institutes of Health, Bethesda, MD, USA).

### RNA pull-down assay

An RNA pull-down assay was conducted to validate the interaction between circ_0003865 and miR-3653-3p using the biotin-LNA-3865 probe (5′-TGGATCTGAATCTGTGTAACT-3′) synthesized by the GENEray company (Shanghai, China) as previously described [26]. Briefly, cultured 293T cells overexpressing circ_0003865 were lysed and incubated with the abovementioned probes for 2 h and “pulled down” with streptavidin-coated magnetic beads (#08014; Sigma Aldrich). After washing three times with PBS, the eluates were analyzed by quantitative PCR method to detect both circ_0003865 and miR-3653-3p. At least three biological replicates were conducted.

### Dual-luciferase reporter assay

The interactions between miR-3653-3p and circ_0003865 or the 3′ UTR region of the GAS1 gene were verified using the Dual-Luciferase Reporter Assay System (Promega) according to the manufacturer’s instructions. Briefly, the circ_0003865 sequences and its mutant version, as well as the GAS1 3′ UTR sequences and its mutant version, were amplified by RT-PCR and were ligated into PmirGLO plasmids. The plasmids were transfected into 293T cells using the Lipofectamine TM 2000 transfection kit (Invitrogen, USA) according to the manufacturer’s instructions, along with miR-3653-3p mimics or a negative control. Subsequently, the cells were lysed using the Passive Lysis Buffer and analyzed using the GloMax-20/20 luminometer (#E5311; Promega). At least three biological replicates were conducted for comparison of luciferase activities between groups.

### Mouse OP model and evaluation

The mouse OP model was established by ovariectomy surgery as previously described [27] and approved by the Experimental Animal Care and Usage Committee of the Sun Yat-sen University (Guangdong, China). The LV5-sh_NC and LV5-sh-hsa_circ_0003865 lentiviruses were packaged and prepared by the Vigene Biosciences company (Jinan, Shandong, China). Thirty female SPF-grade C57BL/6J mice between 6 and 8 weeks old were purchased from Vital River Laboratories (Beijing, China) and randomly divided into a sh-NC group (*n* = 10), an OP + sh-NC group (*n* = 10), and an OP + sh-hsa_circ_0003865 group (*n* = 10). The sh-NC group was injected with 10 μl empty vector at the distal femur (10^13^ copies/mouse) before the sham operation. Mice in the OP + sh-NC group were injected with a 10 μl empty vector (10^13^ copies/mouse) in the distal femur, followed by ovariectomy surgery. Mice in the OP + sh-hsa_circ_0003865 group were injected with 10 μl of sh-hsa_circ_0003865 (10^13^ copies/mouse) in the distal femur and then subjected to ovariectomy. Bone microstructure analysis by micro-CT examination was conducted as previously described [16]. Quantitative RT-PCR was used to detect the relative expression of circ-0003865, *ALP*, *RUNX2*, *OPN*, *GAS1*, and miR-3865-3P in bone tissues. The relative abundance of RFP (Abcam, Cambridge, MA, USA), ALP, RUNX2 OPN, and GAS1 proteins in bone tissues was measured by immunofluorescence using the Immunol Fluorescence Staining Kit (#P0179; Beyotime Biotech) according to the manufacturer’s instructions.

### Statistical analysis

All quantitative data obtained from at least three biological replicates are presented as the mean ± standard deviation in the present study and analyzed using SPSS 20.0 software for evaluating statistical significances. The differences between 2 and > 2 groups were determined by Student’s *t* test and analysis of variance methods, respectively. Significant differences between groups were defined by a *P* value of < 0.05.

## Results

### MEL treatment promotes the osteogenic differentiation of human BMSCs

**Fig. 1:**
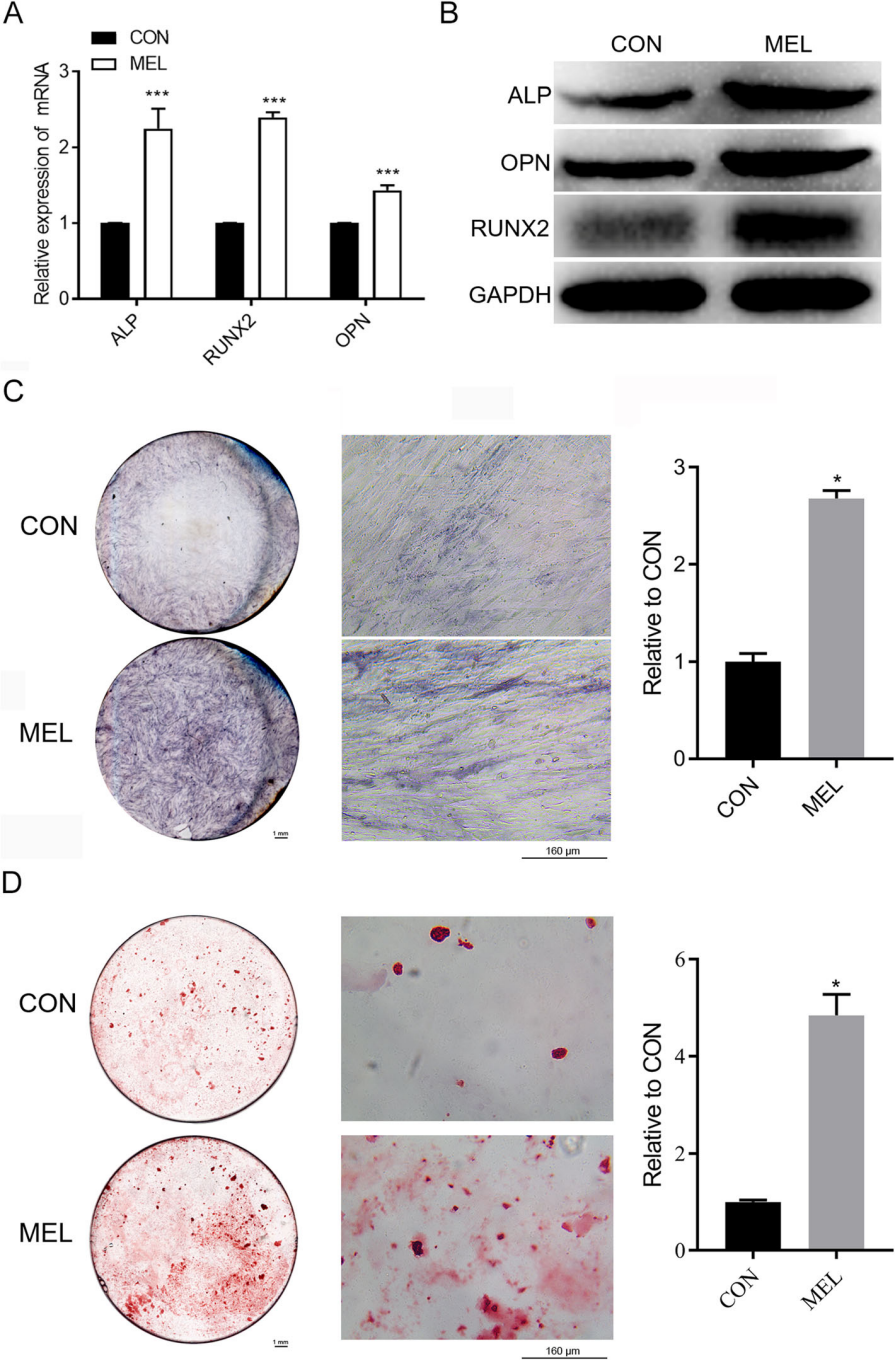
Enhanced osteogenic differentiation of BMSCs by MEL treatment. **a** Relative mRNA levels of osteogenic marker genes in human BMSCs treated with MEL. The *ALP*, *RUNX2*, and *OPN* mRNA levels in BMSCs were measured by qRT-PCR. **b** ALP, RUNX2, and OPN protein levels in human BMSCs treated with MEL. Western blot analysis was used to detect protein levels using GAPDH as the internal standard. **c** Elevated ALP expression in human BMSCs caused by MEL treatment. ALP expression in BMSCs was measured by the ALP staining method. Left are gross scanning images (scale bar: 1 mm), the middle are enlarged images (magnification: × 250, scale bar: 160 μm), and the right is quantification of the left gross scanning images. **d** MEL promotes osteogenic differentiation of human BMSCs. The osteogenic differentiation of BMSCs was evaluated by Alizarin Red staining. Left are gross scanning images (scale bar: 1 mm), the middle are enlarged images (magnification: × 250, scale bar: 160 μm), and the right is quantification of the left gross scanning images. CON: control; MEL: melatonin; ALP: alkaline phosphatase; RUNX2: runt-related transcription factor 2; OPN: osteopontin; GAPDH: glyceraldehyde-3-phosphate dehydrogenase; BMSCs: bone marrow mesenchymal stem cells; *P < 0.05, ***P < 0.001

To elucidate the molecular mechanisms underlying MEL-induced BMSC differentiation toward osteoblasts, we first validated the effects of MEL treatment on the osteoblast differentiation of human BMSCs in vitro. Using quantitative RT-PCR, we showed that the relative mRNA levels of three osteoblast marker genes, *ALP*, *RUNX2*, and *OPN*, were significantly increased by MEL treatment in BMSCs (Fig. 1a). The elevated expression of these three osteogenic biomarkers in BMSCs by MEL treatment was also confirmed by western blot (WB) analysis, which showed increased protein levels of these markers (Fig. 1b). Furthermore, ALP staining revealed significantly elevated expression of ALP in human BMSCs following MEL treatment (Fig. 1c). Finally, we verified that MEL treatment substantially promoted the osteogenic differentiation of human BMSCs, compared with the control group (Fig. 1d) using the Alizarin Red staining method. These results confirmed the role of MEL in stimulating the osteogenic differentiation of BMSCs.

### MEL induces substantial alterations of circRNA profiles in human BMSCs

**Fig. 2:**
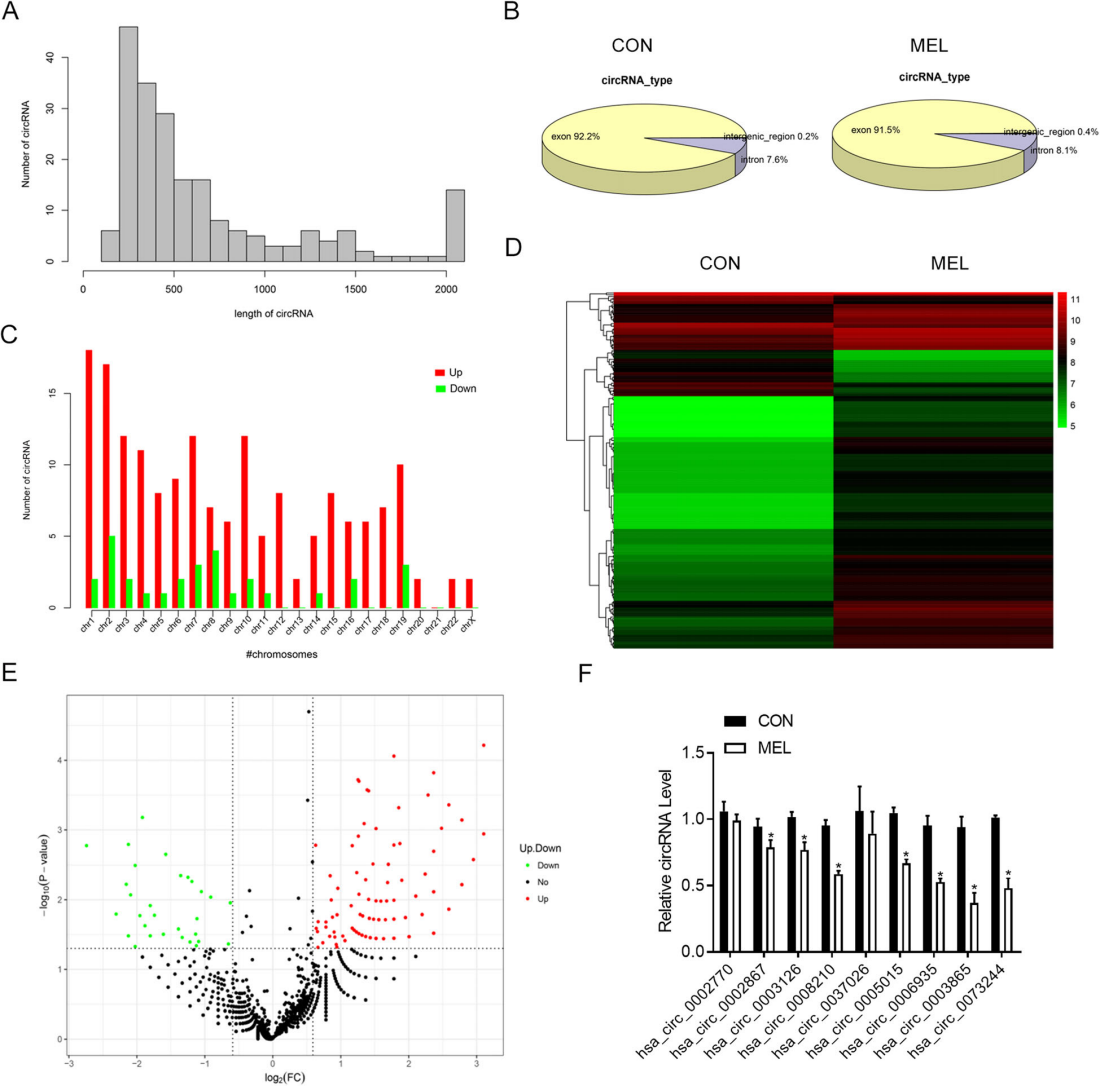
Differential circRNA profiles in MEL-treated human BMSCs. **a** The length distribution of circRNAs identified in human BMSCs treated with MEL. Expression of circRNA profiles in BMSCs was analyzed by deep RNA sequencing. **b** The genomic distribution of circRNAs identified in human BMSCs treated with MEL based on gene exon, intron, and intergenic regions. **c** The numbers of circRNAs encoded by each human chromosome in BMSCs. Upregulated and downregulated circRNAs by MEL treatment are shown in red and green, respectively. **d** Hierarchical clustering of differentially expressed circRNAs in human BMSCs induced by MEL treatment. Differentially expressed circRNAs were defined by a fold change of > 1.5 and a *P* value of < 0.05. The increase and decrease of circRNAs are indicated by red and green colors, respectively. **e** A volcano plot showing the differential expression of circRNAs in BMSCs treated with MEL. Upregulated and downregulated circRNAs are indicated by red and green spots, respectively. Black spots represent CircRNAs with no significant alterations. **f** Relative expressional levels of nine representative differential circRNAs detected by RNA sequencing. The expression of circRNAs was evaluated by qRT-PCR. CON: control; MEL: melatonin; BMSCs: bone marrow mesenchymal stem cells; *P < 0.05

To explore the potential involvement of circRNAs in MEL-induced BMSC osteogenic differentiation, we identified the differentially expressed circRNAs in human BMSCs following MEL treatment or not using a deep RNA sequencing method. A total of 7272 and 5057 circRNAs were detected in the control (BMSC osteogenic differentiation) and MEL treatment groups (BMSC osteogenic differentiation with MEL treatment), respectively. We found that the lengths of most circRNAs identified in human BMSCs were less than 2000 bp, and the majority of circRNAs possessed a length of less than 800 bp (Fig. 2a). Among them, 92.2% and 91.5% of the circRNAs were encoded by exon regions in the control and MEL treatment groups, respectively (Fig. 2b). Furthermore, we found that these circRNAs were distributed to almost every human chromosome, with the largest numbers of upregulated circRNAs encoded by chromosomes 1 and 2, whereas the largest numbers of downregulated circRNAs were encoded by chromosomes 2 and 8 (Fig. 2c). More importantly, we observed that the expression of 209 circRNAs was significantly altered by MEL treatment in human BMSCs, including 173 upregulated and 36 downregulated circRNAs (fold change > 1.5; *P* < 0.05) (Fig. 2d, e). The significantly differentially expressed circRNAs between the control and MEL treatment groups are clearly visualized by hierarchical clustering (Fig. 2d) and a volcano plot (Fig. 2e). Furthermore, we validated the differential expression of nine circRNAs, which may be involved in the osteogenic differentiation of BMSCs in MEL-treated human BMSCs by qRT-PCR (Fig. 2f). A total of seven circRNAs exhibited significant decreased expression in BMSCs following MEL treatment (Fig. 2f). Circ_0003865 exhibited the greatest decrease in expression by MEL consistent with the sequencing results (Fig. 2f). This was further investigated in subsequent experiments.

### MEL significantly alters gene expression profiles in human BMSCs

**Fig. 3:**
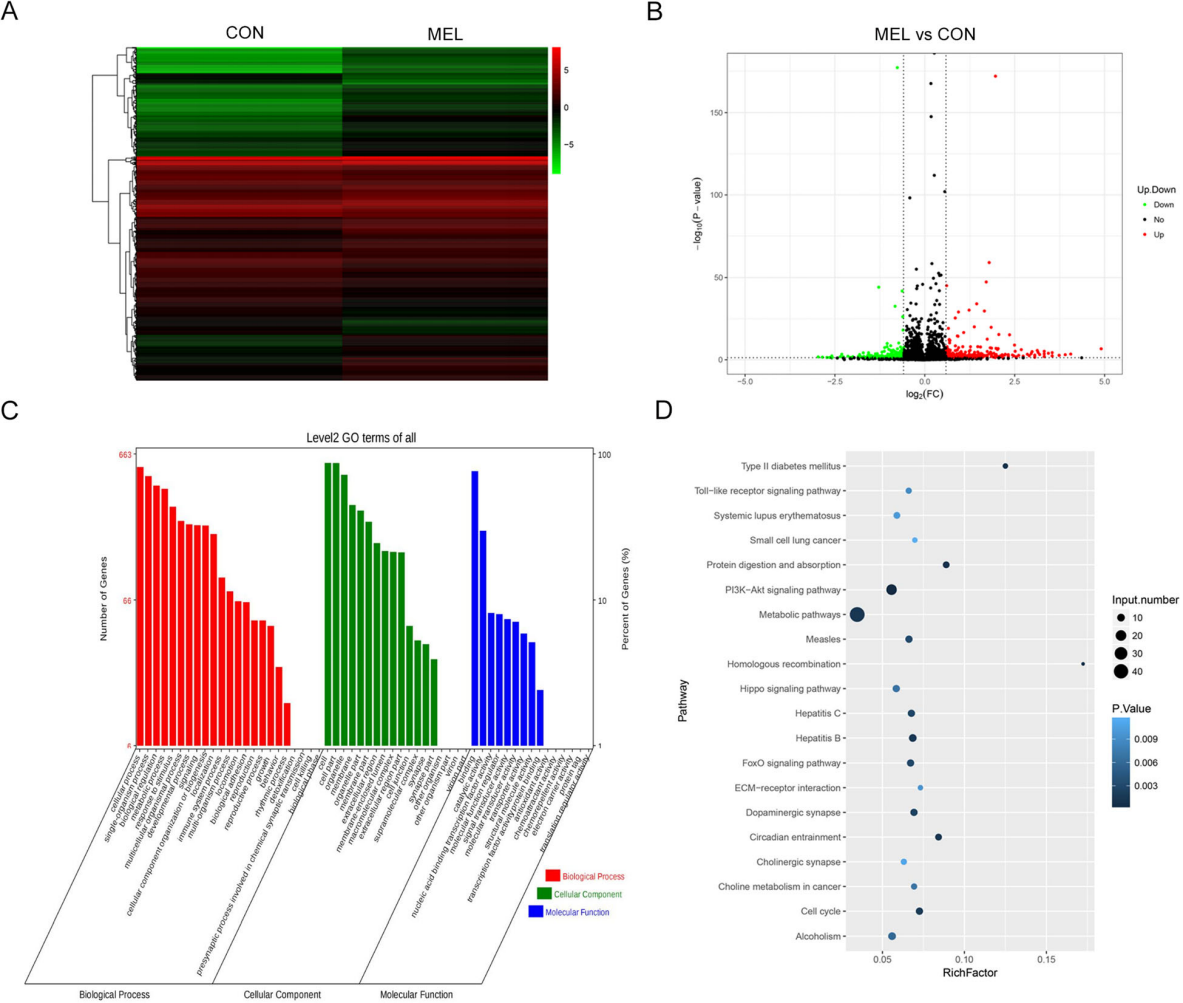
Differential mRNA profiles caused by MEL in human BMSCs. **a** Hierarchical clustering of differentially expressed mRNAs in MEL-treated human BMSCs. Differentially mRNA expression was defined by a fold change of > 1.5 and a *P* value of < 0.05. Red and green colors, respectively, show the increase and decrease of mRNAs. **b** A volcano plot presenting the differentially expressed mRNAs in BMSCs induced by MEL treatment. Upregulated and downregulated mRNAs were represented by red and green spots, respectively. The mRNAs showing no significant expression changes are indicated by black spots. **c** Functional categorization of differentially expressed mRNAs in MEL-treated human BMSCs. The enrichment of differentially expressed mRNAs in biological processes, cellular components, and molecular functions was analyzed by the GO method. **d** Significant enrichment of MEL-induced differentially expressed mRNAs in KEGG signaling pathways. The number of mRNAs in each pathway and the significance of enrichment are indicated by the circle diameters and blue colors, respectively. CON: control; MEL: melatonin; GO: gene ontology; KEGG: Kyoto Encyclopedia of Genes and Genomes

For more insights into the mechanisms underlying BMSC differentiation regulated by MEL, changes in the transcriptomes of BMSCs after MEL treatment were also analyzed by deep RNA sequencing in comparison with the control group. A total of 831 mRNAs showed significant differential expression in human BMSCs induced by MEL treatment, including 513 upregulated and 318 downregulated mRNAs (fold change > 1.5; *P* < 0.05) (Fig. 3a and b). The significant differences in the transcriptomes of BMSCs resulting from MEL treatment were visualized by hierarchical clustering (Fig. 3a) and a volcano plot (Fig. 3b). Using GO categorization, we showed that these differentially expressed genes in MEL-treated BMSCs were mainly enriched in the following biological categories: metabolism processes, responses to stimulus, development process, signaling, cellular component organization or biogenesis, localization, immune system process, reproduction process, rhythmic processes, and detoxification (Fig. 3c). We also found that these differentially expressed genes were associated with various KEGG signaling pathways, such as the metabolism, phosphatidylinositol 3-kinase protein kinase B (PI3K-AKT) signaling, forkhead box O (FoxO) signaling, hippo signaling, extracellular matrix receptor interaction, dopaminergic synapses, cell cycle, and toll-like receptor signaling pathways (Fig. 3d). The significant transcriptional alterations further suggested that the effective regulation of BMSC osteogenic differentiation by MEL might be mediated by complex molecular mechanisms.

### Circ_0003865 knockdown promoted the osteogenic differentiation of BMSCs

**Fig. 4:**
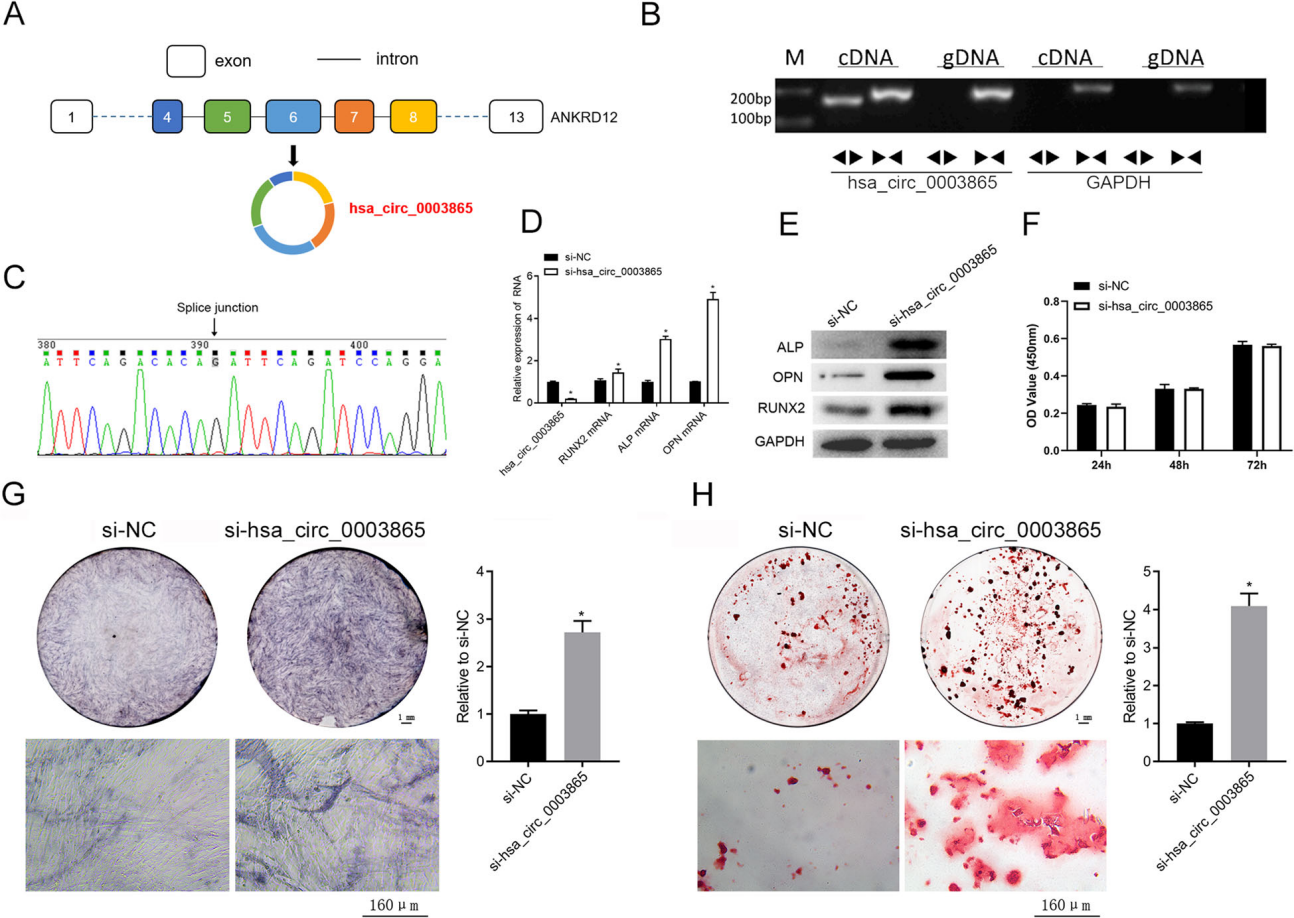
Activation of BMSC osteogenic differentiation by circ_0003865 knockdown. **a** A schematic illustration of circ_0003865 formation from the back-splicing of ANKRD12 gene pre-mRNA. Exons 4–8 of the ANKRD12 gene contributed to the formation of circ_0003865. **b** Detection of circ_0003865 formation in human BMSCs by RT-PCR. The existence of circ_0003865 in human BMSCs was validated by RT-PCR using a divergent primer pair in combination with cDNA samples as the template. Opposite-directed primers and gDNA were used as negative controls. **c** Characterization of the splice junction of circ_0003865 in human BMSCs by Sanger DNA sequencing. The samples amplified by RT-PCR using divergent primers were subjected to Sanger DNA sequencing. **d** Relative expression of circ_0003865 and osteogenic differentiation marker genes in BMSCs transfected with circ_0003865 siRNAs. qRT-PCR was performed to detect circRNA and mRNA expression in BMSCs. **e** ALP, RUNX2, and OPN protein levels in BMSCs transfected with circ_0003865 siRNAs. Protein levels were determined by western blot analysis using GAPDH as the internal standard. **f** No significant change of proliferation in human BMSCs by circ_0003865 knockdown. The CCK-8 assay was used to detect the proliferation in BMSCs. **g** Increased ALP expression in human BMSCs by circ_0003865 knockdown. The ALP staining method was used to measure in BMSCs. The top left are gross scanning images (scale bar: 1 mm), the lower left are enlarged images (magnification: 250×, scale bar: 160 μm), and the right is quantification of the left gross scanning images. **h** The enhanced osteogenic differentiation of human BMSCs by circ_0003865 knockdown. The osteogenic differentiation of human BMSCs was evaluated using the Alizarin Red staining method. The top left are gross scanning images (scale bar: 1 mm), the lower left are enlarged images (magnification: × 250, scale bar: 160 μm), and the right is quantification of the left gross scanning images. si-NC: negative control siRNA; si-hsa_circ_0003865: circ_0003865 siRNA; M: marker; gDNA: genomic DNA; CCK-8: cell counting kit-8; ALP: alkaline phosphatase; RUNX2: runt-related transcription factor 2; OPN: osteopontin; GAPDH: glyceraldehyde-3-phosphate dehydrogenase; *P < 0.05

As shown in Fig. 2f, the expression of circ_0003865 was significantly reduced in human BMSCs treated with MEL. Bioinformatic analysis revealed that the circ_0003865 was produced from the back-splicing of pre-mRNAs encoded by the ankyrin repeat domain 12 (ANKRD12) gene (Fig. 4a). The expression of circ_0003865 in human BMSCs was validated by RT-PCR using divergent primers in BMSC cDNA samples but not in BMSC gDNA (genomic DNA) samples (Fig. 4b). Furthermore, the covalently closed form of circ_0003865 was further confirmed by Sanger sequencing, which identified the splice junction of circ_0003865 amplified and purified from human BMSCs (Fig. 4c). To analyze the role of circ_0003865 in BMSC osteogenic differentiation, its expression in human BMSCs was then effectively knocked down by transfection with specific siRNAs targeting circ_0003865 (Fig. 4d). We observed that the expression of the *RUNX2*, *ALP*, and *OPN* genes in human BMSCs with circ_0003865 knockdown was all significantly higher than those in the si-NC group (Fig. 4d). Consistently, the protein levels of RUNX2, ALP, and OPN in human BMSCs were all substantially elevated by circ_0003865 knockdown (Fig. 4e). Furthermore, we observed that there was no significant effect on proliferation after circ_0003865 knockdown compared to those in si-NC group (Fig. 4f). And the elevated expression of ALP in human BMSCs transfected with circ_0003865 siRNAs was further verified by the ALP staining method (Fig. 4g). Importantly, we showed by Alizarin Red staining that circ_0003865 knockdown resulted in significant enhanced osteogenic differentiation in BMSCs (Fig. 4h). These results indicate that circ_0003865 acts as a negative regulator of BMSC osteogenic differentiation.

### MEL promotes BMSC osteogenic differentiation by suppressing circ_0003865 expression

**Fig. 5:**
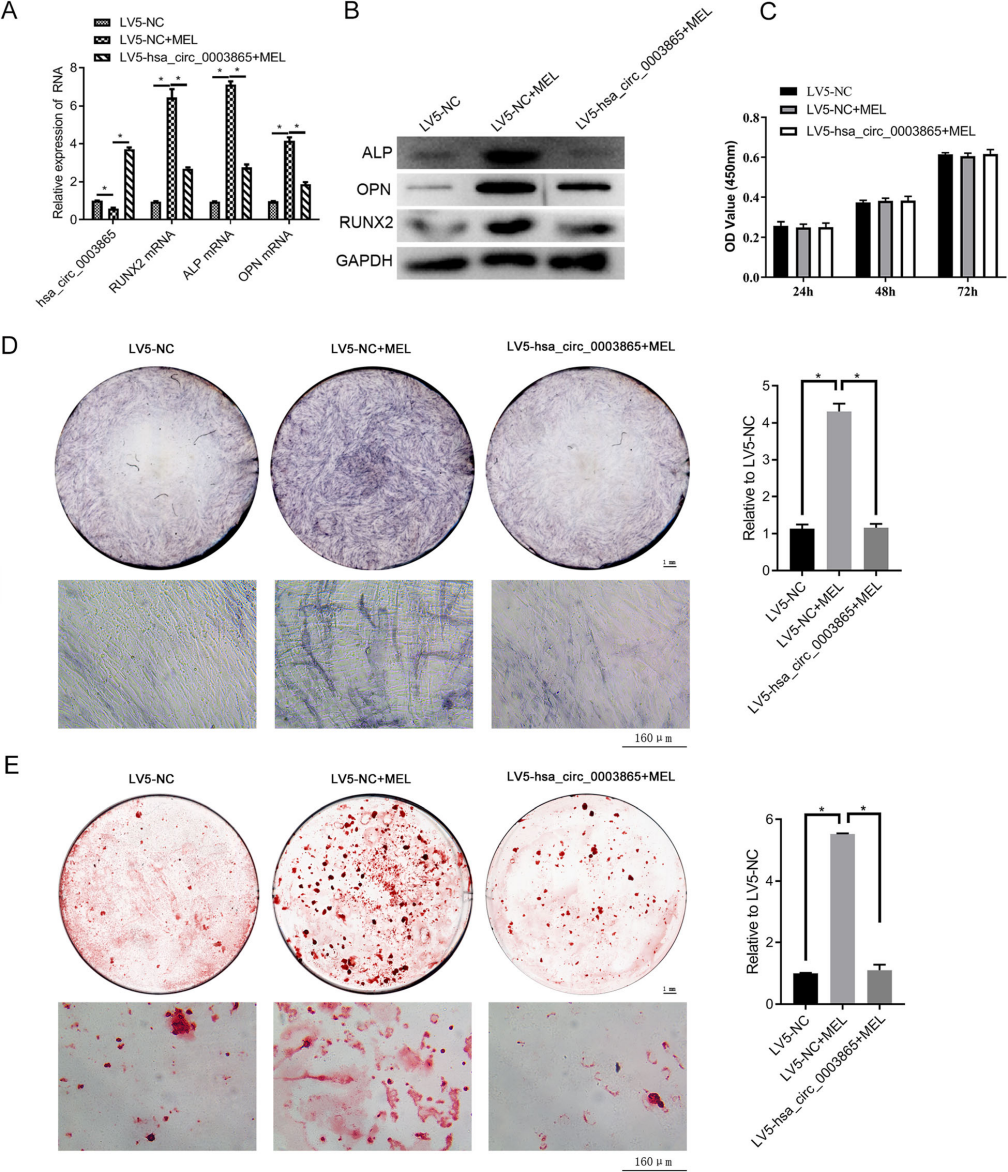
Abrogation of MEL promotes BMSC osteogenic differentiation by circ_0003865 overexpression. **a** Relative expression of circ_0003865 and three osteogenesis marker genes in BMSCs. qRT-PCR was used to measure circRNA and mRNA levels in BMSCs. **b** The relative abundance of ALP, RUNX2, and OPN proteins in BMSCs. Osteogenesis marker protein abundances were determined by western blot analysis with GAPDH as the internal standard. **c** No significant change of proliferation in human BMSCs treated with MEL by circ_0003865 overexpression. CCK-8 assay was used to detect the proliferation. **d** Suppression of ALP expression in human BMSCs treated with MEL by circ_0003865 overexpression. ALP expression in BMSCs was detected by ALP staining. The top left are gross scanning images (scale bar: 1 mm), the lower left are enlarged images (magnification: × 250, scale bar: 160 μm), and the right is quantification of the left gross scanning images. **e** Inhibition of osteogenic differentiation of human BMSCs under MEL treatment by circ_0003865 overexpression. The Alizarin Red staining method was done to detect BMSC osteogenic differentiation. The top left are gross scanning images (scale bar: 1 mm), the lower left are enlarged images (magnification: × 250, scale bar: 160 μm), and the right is quantification of the left gross scanning images. MEL: melatonin; CCK-8: cell counting kit-8; ALP: alkaline phosphatase; RUNX2: runt-related transcription factor 2; OPN: osteopontin; GAPDH: glyceraldehyde-3-phosphate dehydrogenase; *P < 0.05

To address the role of circ_0003865 in MEL-induced BMSC osteogenic differentiation, we then overexpressed circ_0003865 in human BMSCs by infection with the LV5-hsa_circ_0003865 lentivirus. Using qRT-PCR, we initially confirmed that the expression of circ_0003865 was significantly elevated by LV5-circ_0003865 compared with that by the LV5-NC group even with MEL treatment (Fig. 5a). Then, we demonstrated the elevation of *ALP*, *RUNX2*, and *OPN* gene expression in the LV5-NC + MEL (MEL) group compared with that in the LV5-NC group. All of these genes were significantly repressed by circ_0003865 overexpression during MEL treatment (Fig. 5a). These trends were observed in the protein expression of ALP, RUNX2, and OPN as determined by WB analysis (Fig. 5b). Furthermore, CCK-8 assay indicated that circ_0003865 overexpression had no effect on proliferation (Fig. 5c). And ALP staining revealed that circ_0003865 overexpression significantly mitigated the increase of in situ ALP gene expression in BMSCs induced by MEL treatment (Fig. 5d). Similarly, we used the Alizarin Red staining method to demonstrate that the MEL-induced promotion of osteogenic differentiation of human BMSCs was also substantially repressed by circ_0003865 overexpression (Fig. 5e). These assays indicate that the activation of BMSC osteogenic differentiation by MEL is mediated by its inhibition of circ_0003865 expression.

### Circ_0003865 inhibits the expression of miR-3653-3p, which suppresses GAS1 expression and promotes osteogenic differentiation in BMSCs

**Fig. 6:**
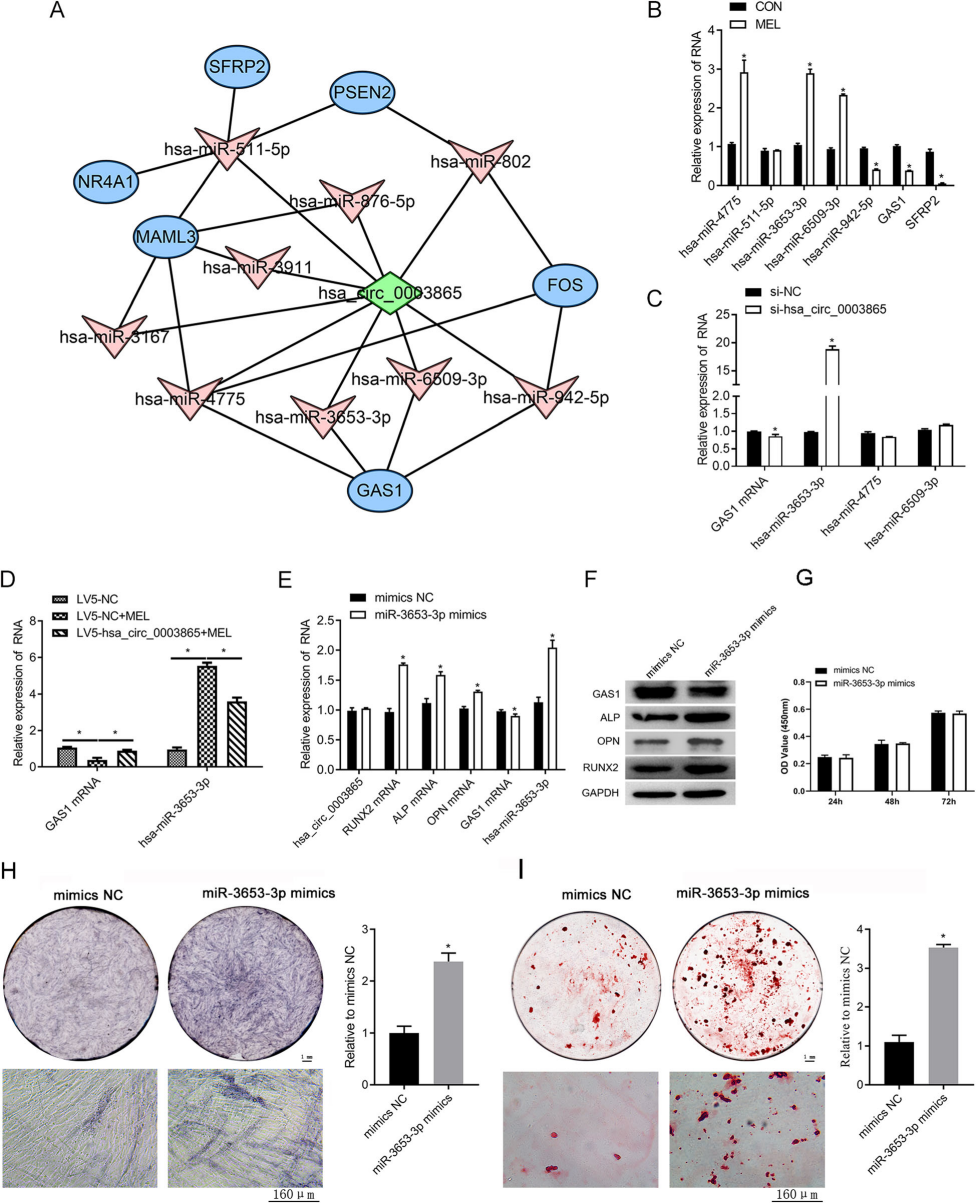
Inhibition of miR-3653-3p expression by circ_0003865 and the effects of miR-3653-3p on GAS1 expression and BMSC osteogenic differentiation. **a** The interaction networks between circ_0003865 with microRNAs and target genes. The microRNAs and target genes were predicted to interact with circ_0003865 using the miRanda database. **b** Relative expression of representative microRNAs and target genes in BMSCs treated with MEL. The expression of microRNAs and mRNAs were detected by qRT-PCR. **c** Elevated miR-3653-3p and decreased GAS1 expression in human BMSCs by circ_0003865 knockdown. Relative expression of microRNAs and GAS1 was measured by qRT-PCR. **d** circ_0003865 abrogates the suppression of GAS1 expression and elevates miR-3653-3p expression in BMSCs caused by MEL treatment. Relative expression of microRNAs and GAS1 was measured by qRT-PCR. **e** Influence of miR-3653-3p overexpression on circ_0003865, GAS1, and osteogenic biomarker gene expression in human BMSCs. Relative expression was evaluated by qRT-PCR. **f** ALP, RUNX2, OPN, and GAS1 protein levels in human BMSCs transfected with miR-3653-3p mimics. Western blot analysis was conducted to measure the protein levels in BMSCs. **g** Proliferation in human BMSCs transfected with miR-3653-3p mimics was detected by CCK-8 assay. **h** In situ expression of ALP in human BMSCs transfected with miR-3653-3p mimics was detected by ALP staining. The top left are gross scanning images (scale bar: 1 mm), the lower left are enlarged images (magnification: × 250, scale bar: 160 μm), and the right is quantification of the left gross scanning images. **i** The enhancement of osteogenic differentiation of human BMSCs by miR-3653-3p mimics. Alizarin Red staining was conducted to evaluate the osteogenic differentiation of human BMSCs. The top left are gross scanning images (scale bar: 1 mm), the lower left are enlarged images (magnification: × 250, scale bar: 160 μm), and the right is quantification of the left gross scanning image. MEL: melatonin; GAS1: growth arrest-specific gene 1; SFRP2: secreted frizzled-related protein 2; CCK-8: cell counting kit-8; ALP: alkaline phosphatase; RUNX2: runt-related transcription factor 2; OPN: osteopontin; NC: negative control; GAPDH: glyceraldehyde-3-phosphate dehydrogenase; *P < 0.05

For insights into the molecular mechanisms downstream of circ_0003865, we subsequently investigated the potential involvement of microRNAs and functional gene expression in BMSC osteogenic regulation by circ_0003865. Using a bioinformatic analysis, circ_0003865 was predicted to target multiple microRNAs and genes (Fig. 6a). Among them, GAS1 and secreted frizzled-related protein 2 (SFRP2) may be involved in the BMSC osteogenic differentiation signaling pathway. These genes were also downregulated in mRNA sequencing after MEL treatment, so we evaluated the two genes and corresponding miRNAs. The results showed that the expression of miR-3653-3p, miR-4775, and miR-6509-3p was significantly elevated by MEL treatment in BMSCs, whereas the expression of GAS1 and SFRP2 was significantly reduced (Fig. 6b). Furthermore, we found that circ_0003865 knockdown resulted in the elevation of miR-3653-3p expression and reduced GAS1 expression in human BMSCs (Fig. 6c). Furthermore, MEL treatment promoted miR-3653-3p expression and suppressed GAS1 expression, and this effect was mitigated by circ_0003865 overexpression (Fig. 6d). These results indicate that circ_0003865 inhibits miR-3653-3p expression and promotes GAS1 expression in human BMSCs.

To further explore the roles of miR-3653-3p in regulating BMSC osteogenic differentiation, we then overexpressed miR-3653-3p in human BMSCs by transfection with specific mimics (Fig. 6e). The overexpression of miR-3653-3p resulted in a significant reduction of GAS1 expression and a marked elevation of ALP, RUNX2, and OPN expression in human BMSCs, but no changes in circ_0003865 expression (Fig. 6e). A concomitant decrease in GAS1 protein levels and an increase in ALP, RUNX2, and OPN protein levels were also detected in BMSCs transfected with the miR-3653-3p mimics (Fig. 6f). Furthermore, we showed by CCK-8 assay that miR-3653-3p mimics had no effect on proliferation (Fig. 6g), and we also showed by ALP staining that the in situ expression of the ALP gene in human BMSCs was significantly enhanced by the miR-3653-3p mimics (Fig. 6h). Additionally, Alizarin Red staining showed that the osteogenic differentiation of human BMSCs were promoted by the miR-3653-3p mimics (Fig. 6i). These results indicate that miR-3653-3p can suppress GAS1 gene expression to promote osteogenic differentiation of BMSCs.

### Circ_0003865 sponges miR-3653-3p to modulate GAS1 expression and BMSC osteogenic differentiation

**Fig. 7:**
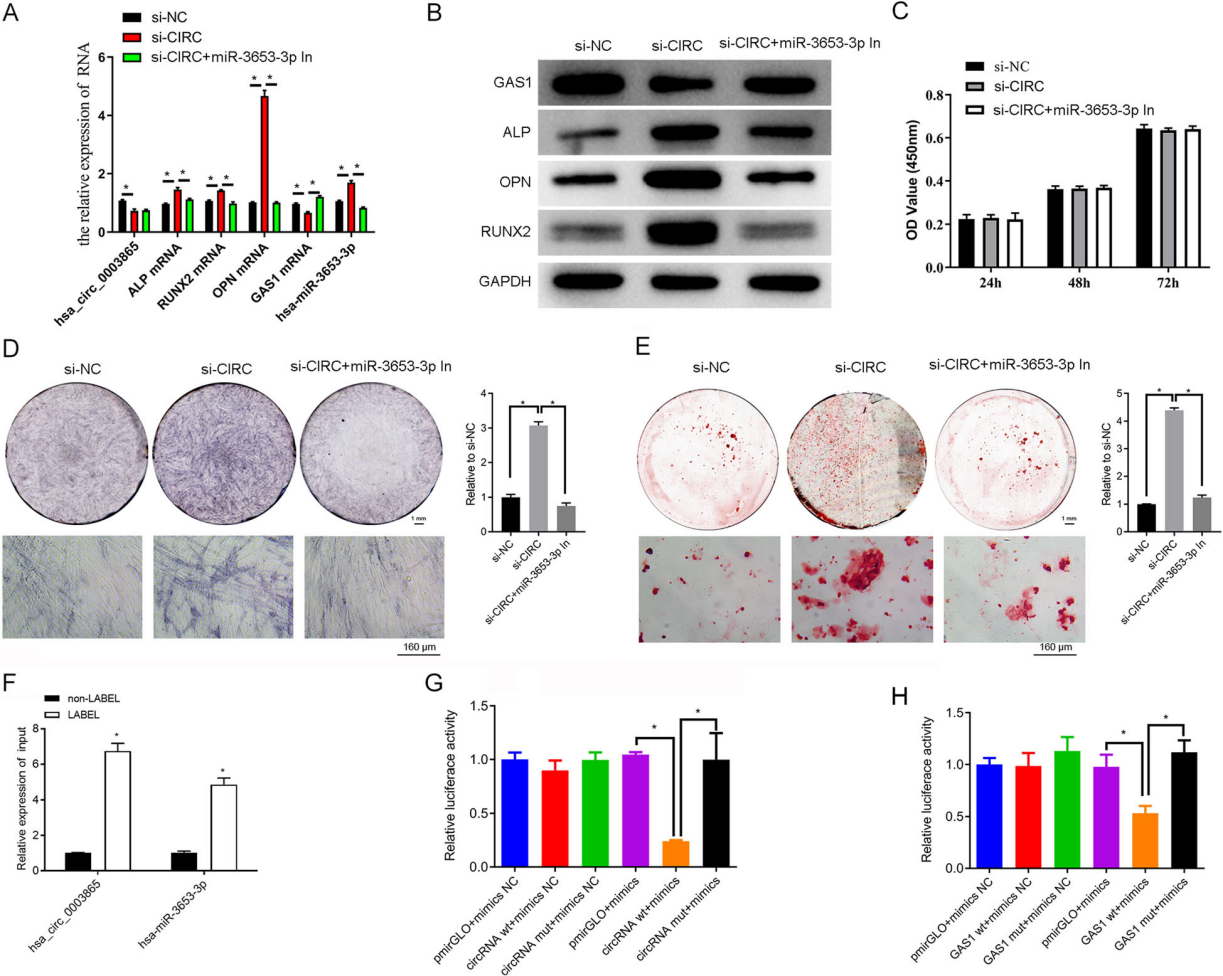
miR-3653-3p reverses circ_0003865-regulated BMSC osteogenic differentiation by directly binding circ_0003865 and the GAS1 gene. **a** Relative expression of circ_0003865, miR-3653-3p, *GAS1*, and osteogenic marker genes in BMSCs as measured by qRT-PCR. **b** GAS1, ALP, RUNX2, and OPN protein levels in human BMSCs as quantitated by western blot analysis using GAPDH as the internal standard. **c** The proliferation in human BMSCs was tested by CCK-8 assay. **d** The expression of ALP in human BMSCs was analyzed by ALP staining. The top left are gross scanning images (scale bar: 1 mm), the lower left are enlarged images (magnification: 250×, scale bar: 160 μm), and the right is quantification of the left gross scanning images. **e** miR-3653-3p inhibitors repressed the osteogenic differentiation of human BMSCs transfected with circ_0003865 siRNAs compared with the si-CIRC group. The Alizarin Red staining was done to evaluate BMSC osteogenic differentiation. The top left are gross scanning images (scale bar: 1 mm), the lower left are enlarged images (magnification: × 250, scale bar: 160 μm), and the right is quantification of the left gross scanning images. **f** The association of circ_0003865 with miR-3653-3p as detected by an RNA pull-down assay. **g**, **h** The direct binding of miR-3653-3p with circ_0003865 and GAS1 gene as measured by the dual-luciferase reporter assay. Between miR-3653-3p and circ_0003865 (**g**). Between miR-3653-3p and GAS1 gene (**h**). si-CIRC: circ_0003865 siRNAs; miR-3653-3p In: miR-3653-3p inhibitors; GAS1: growth arrest-specific gene 1; CCK-8: cell counting kit-8; ALP: alkaline phosphatase; RUNX2: runt-related transcription factor 2; OPN: osteopontin; GAPDH: glyceraldehyde-3-phosphate dehydrogenase; NC: negative control; *P < 0.05

To clarify the mediating roles of miR-3653-3p in circ_0003865-regulated BMSC osteogenic differentiation, we then transfected human BMSCs with the combination of circ_0003865 siRNAs (si-CIRC) and miR-3653-3p inhibitors (miR-3653-3p In). We found that miR-3653-3p inhibitors significantly reduced the expression of miR-3653-3p in si-CIRC+miR-3653-3p In group compared with the si-CIRC group. There is also a significant decrease of ALP, RUNX2, and OPN and increases in GAS1 expression in si-CIRC+miR-3653-3p In group compared with the si-CIRC group. No differences were observed in circ_0003865 expression in si-CIRC+miR-3653-3p In compared with the si-CIRC group (Fig. 7a). Additionally, ALP, RUNX2, and OPN protein levels in BMSCs transfected with circ_0003865 siRNAs were significantly reduced by miR-3653-3p inhibitors and accompanied by an increase of GAS1 protein (Fig. 7b). Furthermore, the proliferation had no significant change after circ_0003865 siRNA and miR-3653-3p inhibitor treatment compared to circ_0003865 siRNA treatment (Fig. 7c). And the in-site expression of ALP in BMSCs transfected with circ_0003865 siRNAs was repressed by miR-3653-3p inhibitors (Fig. 7d). Consistently, the osteogenic differentiation of BMSCs transfected with circ_0003865 siRNAs was significantly suppressed by miR-3653-3p inhibitors as well (Fig. 7e). These results indicate that miR-3653-3p reverses circ_0003865-induced alterations of GAS1 expression and BMSC osteogenic differentiation.

We subsequently used an RNA pull-down assay to show that circ_0003865 was directly associated with miR-3653-3p in human BMSCs (Fig. 7f). The dual-luciferase reporter assay also revealed that circ_0003865 could directly bind to miR-3653-3p in human BMSCs (Fig. 7g). Additionally, we showed that hsa-miR-3653-3p was directly bound to the 3′ UTR region of the GAS1 gene (Fig. 7h). Thus, we have discovered a circ_0003865/miR-3653-3p/GAS1 interaction that substantially modulates the osteogenic differentiation of human BMSCs.

### The short-hairpin RNA-mediated silencing of circ_0003865 expression inhibits OP development in a mouse model

**Fig. 8:**
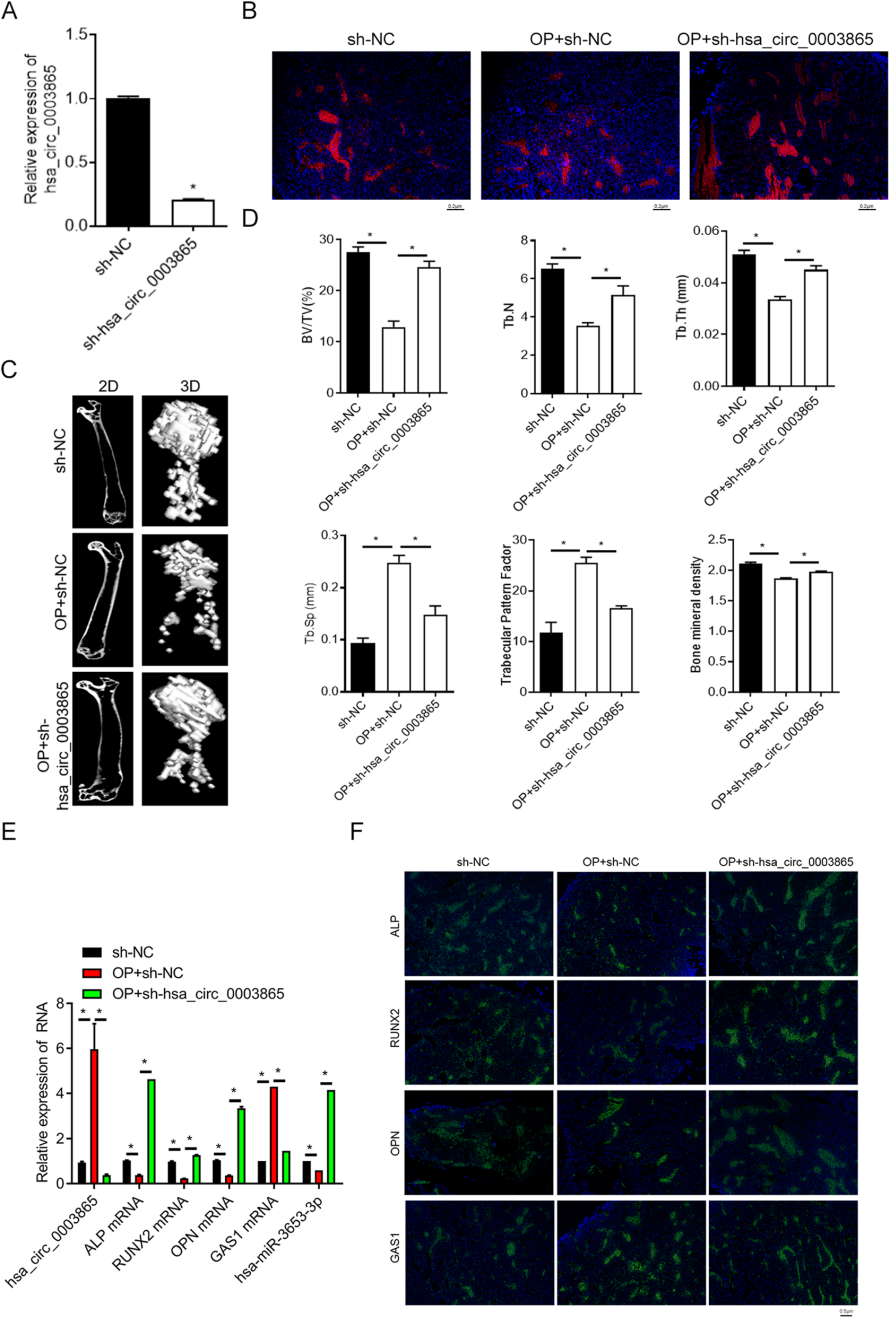
Silencing of circ_0003865 suppresses OP pathogenesis in a mouse model. **a** Decreased circ_0003865 expression in BMSCs caused by infection with adeno-associated virus (AAV) carrying sh-circ_0003865 sequences. Relative expression of circ_0003865 as measured by qRT-PCR. **b** Transfection of circ_0003865-silencing AAVs into bone tissues of the mouse OP model established by ovariectomy. The infection of AAVs in bone tissues was observed by immunofluorescence (magnification: × 200, scale bar: 0.5 μm). **c** Silencing of circ_0003865 restored the femur densities in the mice OP model. Femur densities were analyzed by micro-CT examination. **d** Bone microstructure parameters in the mouse OP model. The Siemens Preclinical Imaging System was used to detect BV/TV, Tb.N, Tb.Th, Tb.Sp, trabecular pattern factor, and bone mineral density of the mouse bones. **e** Relative expression of circ_0003865, miR-3653-3p, *GAS1*, *ALP*, *RUNX2*, and *OPN* mRNAs in bone tissues of the mouse OP model. qRT-PCR was used to measure relative expression. **f** Changes in GAS1, ALP, RUNX2, and OPN protein levels in bone tissues of the mouse OP model. In situ protein expression in mouse bone tissues as detected by immunofluorescence (magnification: × 200, scale bar: 0.5 μm) Sh-NC: negative control; OP: osteoporosis; BV/TV: bone volume/total volume; Tb.N: trabecular number; Tb.Th: trabecular thickness; Tb.Sp: trabecular separation; GAS1: growth arrest-specific gene 1; ALP: alkaline phosphatase; RUNX2: runt-related transcription factor 2; OPN: osteopontin; *P < 0.05

For validation of the roles of circ_0003865 in OP pathogenesis, we finally silenced the expression of circ_0003865 in BMSCs using adeno-associated virus (AAV)-mediated delivery of a short-hairpin RNA targeting circ_0003865. Using BMSCs, we showed that the AAV-delivered sh_circ0003865 effectively silenced the expression of circ_0003865 in human BMSCs compared with BMSCs transfected with sh-NC (negative control)-carrying recombinant AAVs (Fig. 8a). Subsequently, a mouse OP model was established by ovariectomy surgery. The mice were then subjected to circ_0003865 silencing in bone tissues by injecting recombinant AAVs carrying the sh_circ0003865 or sh_NC. Using an immunofluorescence assay, we showed that the mouse bone tissues for the three groups were all successfully transfected with the recombinant AAVs carrying sh_circ0003865 or sh_NC (Fig. 8b). We observed by micro-CT examination that the density of the mouse bilateral femur in the OP + sh-NC group was significantly lower than that in the sh-NC group. Bone density was significantly elevated by sh-circ_0003865 (Fig. 8c). Accordingly, we showed a decrease in bone volume/total volume (BV/TV), trabecular number (Tb.N), trabecular thickness (Tb.Th), and BMD and an increase in Tb.Sp (trabecular separation) and trabecular pattern factor in the OP + sh-NC group compared in the sh-NC group. These effects were reversed significantly by sh_circ_0003865 (Fig. 8d).

Using qRT-PCR, we confirmed the marked elevation of circ_0003865 expression in mouse bone tissue of the OP + sh-NC-treated group compared to that of the sh-NC group. A decrease was observed when circ_0003865 was inhibited (Fig. 8e). In contrast, the expression miR-3653-3p in murine bone tissues was significantly decreased in the OP + sh-NC group compared to that in the sh-NC group. This was also markedly increased by sh-circ_0003865 administration (Fig. 8e). The expression of GAS1 in bone tissues of the OP + sh-NC group was significantly higher than that of the sh-NC group, whereas this effect was downregulated by sh-circ_0003865 administration (Fig. 8e). Furthermore, the relative mRNA levels of ALP, RUNX2, and OPN in bone tissues of the OP + sh-NC group were all significantly lower than those of the sh-NC group, whereas all were markedly elevated by sh-circ_0003865 administration (Fig. 8e). Concomitantly, sh-circ_0003865 induced significant alterations of GAS1, ALP, RUNX2, and OPN protein levels in murine bone tissues between the three groups as determined by immunofluorescence (Fig. 8f). Collectively, these results indicate that the silencing of circ_0003865 suppresses ovariectomy-induced OP in a mouse model by regulating circ_0003865 expression and modulating BMSC osteogenic differentiation.

## Discussion

In recent years, BMSCs have been used for OP treatment because of their potential for differentiating into osteoblasts [6]. Previous studies have revealed that MEL promotes osteogenic differentiation of BMSCs, which indicates that they may be effectively used for BMSC-based OP treatment [14–16]. However, the underlying mechanisms remain largely undefined. In the present study, we focused on the potential role of circRNAs in the MEL-activated osteogenic differentiation of human BMSCs and their association with OP pathogenesis and treatment. We first characterized the significant alterations of circRNA and mRNA expression profiles in BMSCs following MEL treatment by deep RNA sequencing, which was associated with multiple biological processes. Subsequently, circ_0003865 was further shown to be repressed by MEL in BMSCs and sponges miR-3653-3p to modulate *GAS1* gene expression and osteogenic differentiation in human BMSCs. Overexpression of miR-3653-3p promoted BMSC osteogenic differentiation, whereas miR-3653-3p inhibitors abrogated this effect induced by circ_0003865 silencing. Finally, we verified the function of circ_0003865 in repressing BMSC osteogenic differentiation and promoting OP pathogenesis by infecting a murine OP model with AAVs designed to express sh_circ_0003865. These investigations revealed the role of a new circ_0003865/miR-3653-3p/GAS1 signaling axis in MEL-regulated BMSC differentiation and OP treatment.

BMSCs refer to the mesenchymal stem cells derived from the bone marrow. They serve as the progenitors for osteocyte, chondrocytes, and other cell types involved in the formation of skeletal tissues, hematopoiesis-supporting stroma, and adipose tissues [28]. Additionally, BMSCs exhibit a high potential to differentiate into a large spectrum of other specialized human cell types, including cardiac myocytes, neural cells, renal cells, liver hepatocytes, corneal cells, blood cells, and even myogenic cells [28–30]. Benefiting from their multi-faceted differentiation capabilities, high portability, and relatively low immunogenicity, BMSCs have been regarded as ideal candidate stem cells for the treatment of various human disorders over the past decades [31–33]. In adult skeletal tissues, BMSCs primarily differentiate into two cell types: osteoblasts and adipocytes. The biased differentiation of BMSCs toward adipocytes may lead to a decrease of osteoblast cells and OP pathogenesis [6]. Therefore, the various regulatory factors that can promote BMSC osteogenic differentiation may represent potential therapeutic drugs for treating OP [34, 35]. As described above, the indole hormone, MEL, was previously reported to modulate BMSC stemness and osteogenic differentiation [14–16], but little is known about its underlying mechanism(s). In the present study, we first confirmed the role of MEL in enhancing the osteogenic differentiation of human BMSCs. This hormone was then further used to develop a cell model for investigating the molecular mechanisms underlying its role in BMSC differentiation.

Epigenetic events mediated by non-coding RNAs such as circRNAs and microRNAs perform critical roles in the regulation of stem cell fates and differentiation [36, 37]. Although multiple circRNAs are known to mediate the osteogenic differentiation of BMSCs and OP pathogenesis [19–22], the implications of circRNAs in MEL-regulated BMSC differentiation and OP development remain largely unknown, and the MEL on circRNA expression also remain largely unknown as only one study reported that MEL changed the expression of circRNAs in valvular interstitial cells [38]. To study this phenomenon, we conducted a large-scale characterization of differentially expressed circRNAs in human BMSCs following MEL treatment by deep RNA sequencing. The results showed significant alterations of the circRNA profiles in MEL-treated human BMSCs, suggesting a potential role for circRNAs in BMSC differentiation regulation by MEL. It is well known that the major biological functions of circRNAs are mediated by their substantial effects on gene expression [39]. We also identified changes in the gene expressional profiles by RNA sequencing and observed significant expression differences for many functional genes in MEL-treated BMSCs. These were closely associated with various biological processes and signaling pathways, including metabolism, PI3K-AKT, and FOXO signaling cascades. Among them, the FOXO signaling pathway was previously shown to mediate osteogenesis and glucocorticoid-induced OP [21]. Furthermore, the PI3K-AKT signaling pathway has been implicated in the osteogenic differentiation of BMSCs resulting from glucagon-like peptide 1 receptor activation [40]. The significant changes in circRNA and mRNA expression identified in the present study indicate that the regulation of BMSC differentiation by MEL is driven by complex signaling mechanisms. This warrants further investigations and may lead to new discoveries for OP prevention and treatment.

In the present study, circ_0003865 was identified for the first time as a differentially expressed circRNA in MEL-treated human BMSCs. A bioinformatics analysis indicated that circ_0003865 is formed by back-splicing of pre-mRNA encoded by the ANKRD12 gene. Previous reports have shown that ANKRD12 gene expression is correlated with metastasis and poor survival of colorectal cancer patients [41]. Furthermore, the ANKRD12 circRNA modulates the invasiveness of cancer cells [42], but little is known about the role of circRNAs derived from the ANKRD12 gene in BMSC differentiation and OP. We demonstrated that the expression of circ_0003865, encoded by the ANKRD12 gene, was significantly decreased in human BMSCs by MEL treatment. The silencing of circ_0003865 expression increased the expression of osteogenic marker genes, including ALP, RUNX2, and OPN and resulted in the marked induction of osteogenic differentiation. Furthermore, overexpression of circ_0003865 effectively abrogated the MEL-induced expression of osteogenic marker genes and BMSC osteogenic differentiation. This validates the mediating role of circ_0003865 in MEL-induced BMSC differentiation. Finally, we verified that circ_0003865 silencing significantly elevated the expression of osteogenic marker gene expression and repressed the progression of OP in a murine model. This convincingly establishes circ_0003865 as a potent inhibitor of BMSC osteogenic differentiation and a potential biomarker for OP diagnosis and treatment.

Previous reports demonstrated that the gene expression-regulating functions of circRNAs are mainly mediated by targeting miRNAs as sponges to modulate gene expression [43, 44]. We also predicted by a bioinformatics analysis that circ_0003865 sponges miR-3653-3p, miR-4775, and miR-6509-3p to regulate the expression of the *GAS1* gene. The expression of all three microRNAs was significantly increased by MEL treatment in human BMSCs, but only the expression of miR-3653-3p was increased by circ_0003865 silencing. This suggests that miR-3653-3p is a specific target of circ_0003865 in human BMSCs. Furthermore, circ_0003865 overexpression promoted the expression of miR-3653-3p in human BMSCs treated with MEL. Additionally, we showed that miR-3653-3p overexpression greatly elevated ALP expression, RUNX2, and OPN and promoted the osteogenic differentiation of BMSCs. More importantly, miR-3653-3p inhibitors significantly mitigated the circ_0003865 silencing-induced promotion of BMSC osteogenic differentiation, which validates the mediating role of miR-3653-3p in circ_0003865-regulated BMSC differentiation. GAS1 is an inhibitor of the G0 to S phase transition during cell cycle progression and regulates the Hedgehog concentration gradient and related signaling pathways [45, 46] that also suppress muscle stem cell renewal [22]. In the present study, the first time we showed that the GAS1 expression in BMSCs could be suppressed by MEL treatment, enhanced by circ_0003865, and repressed by miR-3653-3p. We also confirmed the direct association between circ_0003865 and miR-3653-3p and the binding of miR-3653-3p with *GAS1* gene sequences using a luciferase reporter assay. This demonstrates the existence of a novel circ_0003865/miR-3653-3p/GAS1 axis in MEL-regulated BMSC osteogenic differentiation.

## Conclusion

In summary, we discovered that MEL enhanced the osteogenic differentiation of human BMSCs and suppressed OP development by inhibiting the expression of circ_0003865, which sponges miR-3653-3p to activate *GAS1* gene expression and repress the expression of osteogenic marker genes. These investigations bring new perspectives for the molecular mechanisms of MEL-induced BMSC osteogenic differentiation and OP inhibition. They will serve as a basis for the clinical use of MEL in treating OP and may reveal new biomarkers for OP diagnosis and treatment.

## Data Availability

The datasets used and/or analyzed during the current study are available from the corresponding author on reasonable request.
